# Regulation of the CoA Biosynthetic Complex Assembly in Mammalian Cells

**DOI:** 10.3390/ijms22031131

**Published:** 2021-01-24

**Authors:** Jovana Baković, David López Martínez, Savvas Nikolaou, Bess Yi Kun Yu, Maria-Armineh Tossounian, Yugo Tsuchiya, Christopher Thrasivoulou, Valeriy Filonenko, Ivan Gout

**Affiliations:** 1 Department of Structural and Molecular Biology, University College London, London WC1E 6BT, UK; jovana.bakovic.16@ucl.ac.uk (J.B.); david.lopez.martinez@crick.ac.uk (D.L.M.); savvasnicolaou93@gmail.com (S.N.); bess.yu.15@ucl.ac.uk (B.Y.K.Y.); m.tossounian@ucl.ac.uk (M.-A.T.); y.tsuchiya115@gmail.com (Y.T.); 2 Research Department of Cell and Developmental Biology, The Centre for Cell and Molecular Dynamics, University College London, London WC1E 6BT, UK; c.thrasivoulou@ucl.ac.uk; 3 Institute of Molecular Biology and Genetics, National Academy of Sciences of Ukraine, 03680 Kyiv, Ukraine; filonenko@imbg.org.ua

**Keywords:** coenzyme A, oxidative stress, extracellular stimuli, coenzyme A biosynthesis

## Abstract

Coenzyme A (CoA) is an essential cofactor present in all living cells. Under physiological conditions, CoA mainly functions to generate metabolically active CoA thioesters, which are indispensable for cellular metabolism, the regulation of gene expression, and the biosynthesis of neurotransmitters. When cells are exposed to oxidative or metabolic stress, CoA acts as an important cellular antioxidant that protects protein thiols from overoxidation, and this function is mediated by protein CoAlation. CoA and its derivatives are strictly maintained at levels controlled by nutrients, hormones, metabolites, and cellular stresses. Dysregulation of their biosynthesis and homeostasis has deleterious consequences and has been noted in a range of pathological conditions, including cancer, diabetes, Reye’s syndrome, cardiac hypertrophy, and neurodegeneration. The biochemistry of CoA biosynthesis, which involves five enzymatic steps, has been extensively studied. However, the existence of a CoA biosynthetic complex and the mode of its regulation in mammalian cells are unknown. In this study, we report the assembly of all five enzymes that drive CoA biosynthesis, in HEK293/Pank1β and A549 cells, using the in situ proximity ligation assay. Furthermore, we show that the association of CoA biosynthetic enzymes is strongly upregulated in response to serum starvation and oxidative stress, whereas insulin and growth factor signaling downregulate their assembly.

## 1. Introduction

Coenzyme A (CoA) is a universal metabolic cofactor essential for the viability of all living cells. It was discovered in the middle of the last century, and numerous anabolic and catabolic pathways have since been found to involve CoA, including the biosynthesis of amino acids, cholesterol, and acetylcholine, the citric acid cycle, synthesis and oxidation of fatty acids, protein acylation, and others [1,2]. These widespread functions of CoA stem from its unique chemical structure, which allows it to act as an acyl group carrier or a carbonyl-activating group by forming diverse derivatives through thioester-linkage. These include central metabolites such as acetyl-CoA, β-hydroxy β-methylglutaryl-CoA (HMG-CoA), malonyl-CoA, and propionyl-CoA. A new post-translational modification (PTM) on proteins, involving CoA (termed protein CoAlation), was recently described in mammalian and bacterial cells, and revealed a novel unconventional function of CoA as an antioxidant in the oxidative and metabolic stress response [3,4,5,6]. The development of novel reagents and methodologies was crucial for demonstrating that protein CoAlation is a widespread and reversible PTM, which occurs in prokaryotic and eukaryotic cells. Protein CoAlation was shown to modulate the activity and conformation of modified proteins, and to protect cysteine residues oxidized to the sulfenic acid state from irreversible overoxidation, under prolonged oxidative and metabolic stress [7,8,9,10]. These advances provided a novel insight into the function of CoA as an important cellular antioxidant in response to oxidative and metabolic stress.

**Figure 1:**
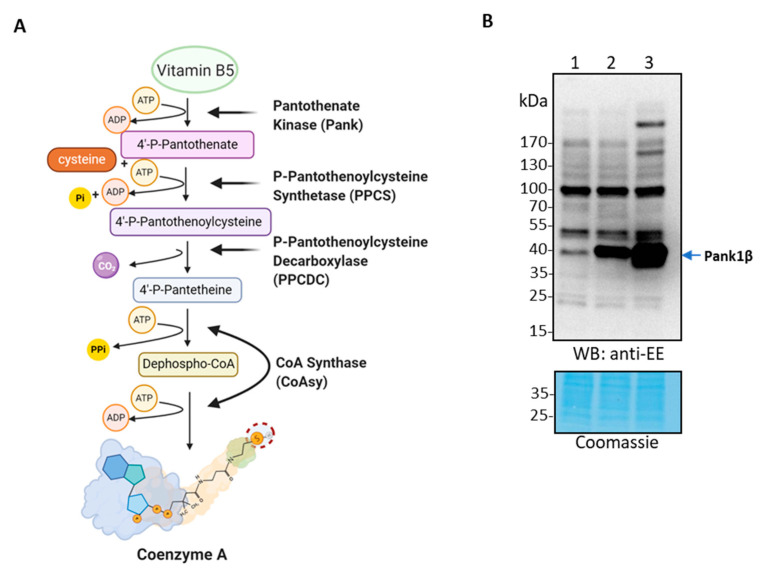
Generation and analysis of HEK293 cell lines with stable overexpression of EE-tagged Pank1β using lentiviral expression system. (**A**) Schematic diagram of the coenzyme A (CoA) biosynthetic pathway in mammalian cells. (**B**) Western blot analysis of EE-Pank1β expression in parental (HEK293) and stable (HEK293/Pank1β) cell lines. Lane 1—HEK293 cells infected with pLex alone (HEK293/pLex); Lane 2—stable HEK293/Pank1β cell line generated by selection for puromycin resistance of cells infected with pLex/Pank1β; Lane 3—transient overexpression of Pank1β in parental HEK293 cells by transfection with pLex/Pank1β, used as positive control. Figure is representative of three independent experiments.

CoA is composed of phosphoadenosine-diphosphate (ADP) and a pantetheine tail containing a highly reactive thiol group, and both moieties are essential for the intracellular functions of CoA. The biosynthesis of CoA is conserved among eukaryotes and prokaryotes and requires five enzymatic reactions (Figure 1A). Pantothenate (vitamin B5, acquired from the diet) is first phosphorylated by pantothenate kinase (Pank) to form 4′-phosphopantothenate. Then, 4′-phosphopantothenoylcysteine synthetase (PPCS) catalyzes the nucleotide-dependent ligation between 4′-phosphopantothenate and cysteine to generate 4′-phosphopantothenoylcysteine. This product then undergoes a decarboxylation reaction to produce 4′-phosphopantetheine, which is catalyzed by 4′-phosphopantothenoylcysteine decarboxylase (PPCDC). The next step involves the transfer of the AMP moiety of ATP by 4′-phosphopantetheine adenylyltransferase (PPAT), forming dephospho-CoA. Finally, dephospho-CoA is phosphorylated by dephospho-CoA kinase (DPCK) at its 3′-hydroxyl group to generate CoA [11]. In mammals, both PPAT and DPCK activities are part of a unique bifunctional enzyme called CoA synthase (CoAsy). Similarly, PPCS and PPCDC in bacteria were found to form a bifunctional enzyme CoaBC [12]. An alternative pathway for de novo CoA biosynthesis has been shown to occur in conditions of impaired Pank activity, mediated by the conversion of extracellular pantethine to CoA by the last two enzymatic steps of the canonical CoA biosynthetic pathway [13].

The biosynthesis of CoA is regulated at multiple levels. The enzymatic feedback mechanism is the main point of regulation and involves the inhibition of Pank, the first rate-limiting enzyme in CoA biosynthesis, by acetyl-CoA, a potent allosteric inhibitor of all four mammalian Pank isoforms [14]. CoA biosynthesis is also regulated via signal transduction pathways and stress response [15,16,17]. Furthermore, studies have shown that PPAT and DPCK activities of CoAsy are strongly induced by phospholipids [18]. Total CoA levels (CoA and its derivatives) were shown to be reduced in response to insulin, glucose, pyruvate and fatty acids, whereas glucagon, glucocorticoids and oxidative stress lead to an increase [19,20,21]. Finally, the total cellular CoA content is also controlled by CoA degradation, involving phosphodiesterases, phosphatases and pantetheinases [22]. Dysregulation of CoA biosynthesis is associated with various human pathologies, including diabetes, cancer, and cardiac hypertrophy [23,24]. Mutations in the human *PANK2* and *COASY* genes were shown to be involved in neurodegeneration with brain iron accumulation (NBIA) [25,26]. Moreover, a recent study showed that mutations in PPCS cause dilated cardiomyopathy [27].

The biochemistry of the CoA biosynthetic pathway has been extensively studied; however, the organization of the CoA biosynthetic machinery remains to be investigated. While the formation of a CoA biosynthetic complex has not been reported in bacteria, a study in yeast identified a multienzyme complex responsible for CoA biosynthesis [28]. It was shown that in *Saccharomyces cerevisiae*, the CoA biosynthetic enzymes (with the exception of Pank) interact to form a CoA-synthesizing protein complex (CoA-SPC), which promotes CoA biosynthesis. Cab3 (analogous to mammalian PPCDC) was identified as the scaffold of this protein complex, but further studies elucidating the regulatory mechanisms of its formation in response to various stimuli are necessary for a better understanding. To date, the existence of a CoA biosynthetic complex and the mode of its regulation in mammalian cells have not been described.

We report the assembly and regulation of the CoA biosynthetic machinery in mammalian cells. Using the proximity ligation assay (PLA), we show that the enzymes of CoA biosynthesis Pank1 and CoAsy, Pank1 and PPCS, and Pank1 and PPCDC, interact in exponentially growing cells. Concurrent with published studies which showed an increase in CoA levels in cells and tissues in response to stresses, we found that oxidative stress and serum starvation strongly induce the association of CoA biosynthetic enzymes in HEK293/Pank1β cells and the A549 lung cancer cell line. We also demonstrate that treatment of cells with insulin and fibroblast growth factor 2 (FGF-2) promotes the dissociation of CoA biosynthetic enzymes in a time-dependent manner. This is the first study reporting the mode by which extracellular stimuli and stresses regulate the association or dissociation of enzymes involved in the universally conserved pathway for CoA biosynthesis. The findings presented in this study open doors for further investigations on the mode of interaction, structural organization, regulation, and subcellular localization of the potential CoA biosynthetic complex in mammalian cells.

## 2. Results

### 2.1. Analysis of the CoA Biosynthetic Complex in HEK293/Pank1β Cells Using Conventional Approaches

Established cell lines contain significantly lower levels of CoA compared to those found in tissues or primary cell lines, indicating lower activity of the CoA biosynthetic enzymes [3]. This phenomenon is most likely due to prolonged culturing of established cell lines in media containing high levels of glucose, which promotes glycolysis at the expense of oxidative phosphorylation and CoA biosynthesis. We therefore reasoned that it would be disadvantageous to use an established cell line for studying the potential assembly between the enzymes of CoA biosynthesis. Overexpression of Pank1β in Cos7 cells was shown to significantly increase the intracellular CoA content [29], and HEK293/Pank1β cells have been previously generated in our laboratory and shown to contain approximately six times more CoA than parental HEK293 cells, which is comparable to CoA levels found in primary cells or tissues [3]. The presence of higher CoA levels suggests higher activity of the enzymes of CoA biosynthesis, and therefore an increased probability of observing a potential CoA biosynthetic complex formation.

In this study, HEK293 cells were infected with a generated lentivirus for stable expression of Pank1β. The expression of EE-tagged Pank1β was analyzed by anti-EE Western Blot (WB) in HEK293 cells transfected with pLex vector alone (Figure 1B, Lane 1), in the HEK293/Pank1β stable cell line generated by infection with the pLex/Pank1β lentivirus construct followed by puromycin selection (Figure 1B, Lane 2), and in HEK293 cells transiently transfected with the pLex/Pank1β lentivirus construct (Figure 1B, Lane 3). The results show that Pank1β expression is significantly increased in the generated stable cell line when compared to cells infected with the pLex vector alone, whereas transient transfection of HEK293 cells with the pLex/Pank1β vector induces the highest increase in Pank1β expression.

Our efforts were initially focused on investigating the existence of a CoA biosynthetic complex in mammalian cells by conventional methods, including co-immunoprecipitation (co-IP) and gel filtration analysis of protein extracts from cell lines or rat tissues under various experimental conditions. In cell-based studies, exponentially growing HEK293/Pank1β cells were examined, with and without the induction of oxidative or metabolic stress. Cell lysates were immunoprecipitated with anti-Pank1, anti-CoAsy, or anti-EE antibodies and the immune complexes were analyzed by WB for the presence of CoAsy, PPCS, and PPCDC. However, our extensive efforts to co-IP other enzymes of the CoA biosynthetic pathway with Pank1 under various experimental conditions were unsuccessful. We next aimed to identify the enzymes of CoA biosynthesis by WB analysis of gel filtration fractions from HEK293/Pank1β cells or rat tissue lysates at the molecular weights corresponding to the potential complex. This approach also proved futile and failed to provide evidence for the presence of enzymes of the CoA biosynthetic pathway in fractions containing high molecular weight proteins or complexes. The dissociation of regulatory protein complexes as a result of cell lysis is a common phenomenon, particularly when their formation involves weak regulatory interactions. We theorize that the formation of a CoA biosynthetic complex is a dynamic and complex process, controlled by signaling and metabolic pathways. Therefore, the loss of cellular integrity and the presence of detergents in the lysis buffer could disrupt regulatory interactions of the CoA biosynthetic complex. This might explain the failed gel-filtration and co-IP approaches, both of which require cell lysis. In addition, the last two steps of CoA biosynthesis are catalyzed by CoAsy, which is present in different cellular compartments (cytoplasm, nucleus, mitochondrial matrix) and was also found to be anchored to the outer mitochondrial membrane (OMM) via its hydrophobic N-terminus [18,26,30,31]. Extracting CoAsy from the OMM during cell lysis may cause significant conformational changes, leading to the dissociation of the CoA biosynthetic complex.

### 2.2. Validation of the Proximity Ligation Assay for Testing the Interaction between Enzymes of CoA Biosynthesis

The PLA assay is a well-established technique for in situ detection of protein–protein interactions [32], and it overcomes the limitations imposed by other methods since it does not require cell lysis. This technique is based on the recognition of target proteins by pairs of antibody–oligonucleotide conjugates (PLA probes). When in close proximity (<40 nm), the oligonucleotides are ligated to form DNA circles that serve as a template for an amplification reaction using complementary fluorogenic oligonucleotides (Supplementary Figure S1).

**Figure 2:**
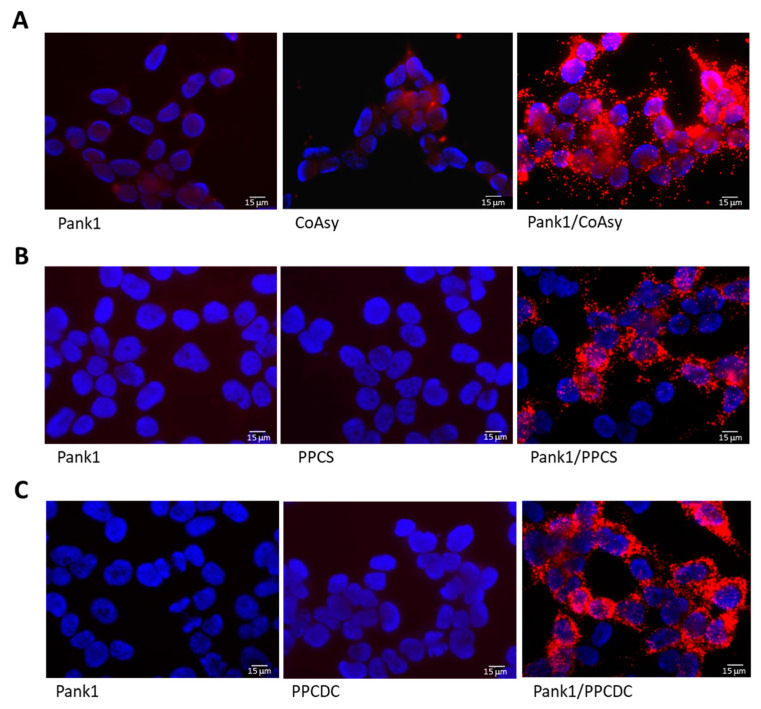
Validation of the proximity ligation assay (PLA) on HEK293/Pank1β cells for the detection of proximity between Pank1 and other enzymes of the CoA biosynthetic pathway. The associations of Pank1 with CoAsy (**A**), PPCS (**B**) and PPCDC (**C**) are shown in exponentially growing HEK293/Pank1β cells. Nuclei stained with DAPI (blue); PLA performed for Pank1 and other enzymes of the CoA biosynthesis (Red). Scale bars: 15 μm. Data are representative of three independent experiments.

To validate the PLA methodology for detecting the interactions between enzymes of the CoA biosynthetic machinery, we examined the association of Pank1 with CoAsy, PPCS, and PPCDC in exponentially growing HEK293/Pank1β cells (Figure 2). HEK293/Pank1β cells were seeded onto coverslips and grown for 24 h in full medium (Dulbecco’s Modified Eagle’s Medium (DMEM), 10% fetal bovine serum (FBS), and 1% penicillin/streptomycin (P/S)). Cells were fixed and the PLA assay was performed according to the manufacturer’s protocol. Briefly, cells were incubated with primary anti-Pank1, anti-CoAsy, anti-PPCS, or anti-PPCDC antibodies, followed by incubation with PLA probes, then ligase, and finally with DNA polymerase (Supplementary Figure S1). The coverslips were dried overnight, mounted with medium containing 4′,6-diamidino-2-phenylindole (DAPI), and cells were visualized with a fluorescent microscope. The PLA images in Figure 2 show that anti-Pank1, anti-CoAsy, anti-PPCS, and anti-PPCDC antibodies alone do not exhibit non-specific background reactivity, as no fluorescent signal is observed. Performing the PLA assay in the presence of both anti-Pank1 and anti-CoAsy (Figure 2A), anti-Pank1 and anti-PPCS (Figure 2B), or anti-Pank1 and anti-PPCDC antibodies (Figure 2C) results in the appearance of readily detectable red fluorescence around the nuclei of most cells, indicating that these enzymes are in close proximity within exponentially growing HEK293/Pank1β cells.

### 2.3. Oxidative Stress and Serum Starvation Induce the Association of CoA Biosynthetic Enzymes

It has been previously shown that CoA biosynthesis is upregulated in response to oxidative stress or fasting, while growth factor signaling and nutrient availability have the opposite effect [19,20,21]. To understand the regulation of the CoA biosynthetic machinery, we explored the association of the CoA biosynthetic enzymes in response to cellular stresses. Culturing cells in serum-free medium is a widely used method in cell and molecular biology for studying signaling pathways and regulatory interactions. Serum starvation was found to induce the production of reactive oxygen species [33,34]. On the other hand, hydrogen peroxide (H_2_O_2_) is a commonly used reagent for the induction of oxidative stress in cell-based models. Serum starvation followed by H_2_O_2_ treatment has also been used to prime cells to oxidative stress [34].

**Figure 3:**
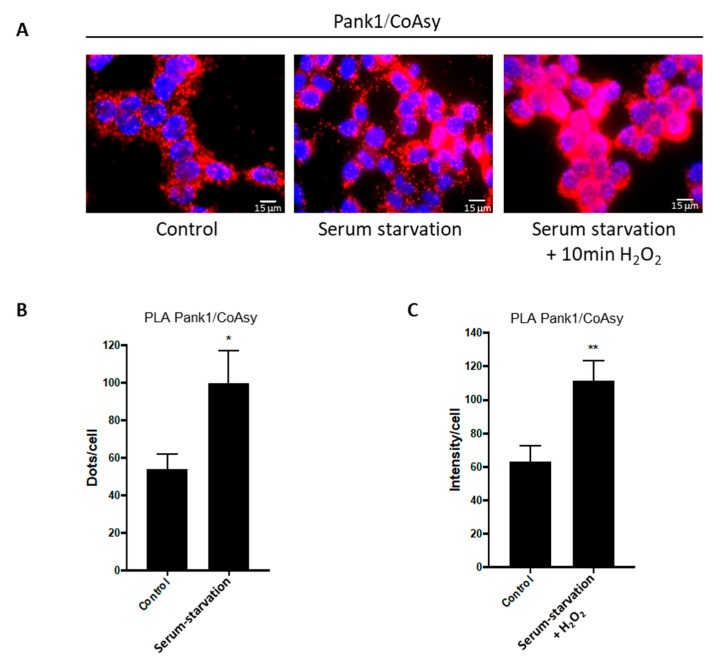
Serum starvation and treatment of HEK293/Pank1β cells with hydrogen peroxide (H_2_O_2_) induce the Pank1/CoAsy association in situ. (**A**) Pank1 interaction with CoAsy is significantly increased in HEK293/Pank1β cells in response to serum starvation (16 h) or serum starvation (16 h) followed by treatment with 500 μΜ H_2_O_2_ (10 min). Nuclei are stained with DAPI (blue); proximity ligation assay (PLA) performed for Pank1 and CoAsy (Red). Scale bars: 15 μm. Images are representative of three independent experiments. (**B**) Relative quantitation of Pank1/CoAsy assembly in control untreated and 16 h serum-starved HEK293/Pank1β cells from (**A**). Dots/cell counted. Data are presented as mean ± standard error of the mean (SEM) from *n* = 3 experiments (* *p* < 0.05). (**C**) Relative quantification of the mean fluorescence intensity per cell for control untreated and serum starved + H_2_O_2_ treated cells from (**A**). Data represent mean ± SEM from *n* = 3 experiments (** *p* < 0.01).

Here, cells were seeded on coverslips and cultured in full DMEM medium for 24 h to achieve ~80% confluency. The media were then replaced with serum-free DMEM, and cells were incubated for another 16 h to induce starvation stress. To explore the synergistic effects of serum starvation and H_2_O_2_-induced stress, cells were treated with 500 μM H_2_O_2_ for 10 min after the 16 h incubation period in serum-free medium. Control cells were grown in full medium and were not treated with H_2_O_2_. The PLA assay was then performed using anti-Pank1 and anti-CoAsy antibodies, and the resulting fluorescence was visualized on a fluorescent microscope (Figure 3). The results show that Pank1 and CoAsy, which mediate the first and last steps of the CoA biosynthetic pathway, interact in exponentially growing HEK293/Pank1β cells, confirming our previous finding (Figure 2A).

Furthermore, serum starvation significantly enhances the association between Pank1 and CoAsy, and the signal is markedly stronger after serum starvation followed by H_2_O_2_ treatment (Figure 3). The fluorescent signal intensities were initially quantified as dots per cell (Figure 3B); however, treating cells with H_2_O_2_ after serum starvation resulted in saturation of the signal, forcing us to express the results as fluorescence intensity per cell (Figure 3C). Overall, both treatments induce an approximate 2-fold increase in the signal intensity compared to control cells.

### 2.4. Treatment of Serum-Starved Cells with Insulin Promotes the Dissociation of CoA Biosynthetic Enzymes

**Figure 4:**
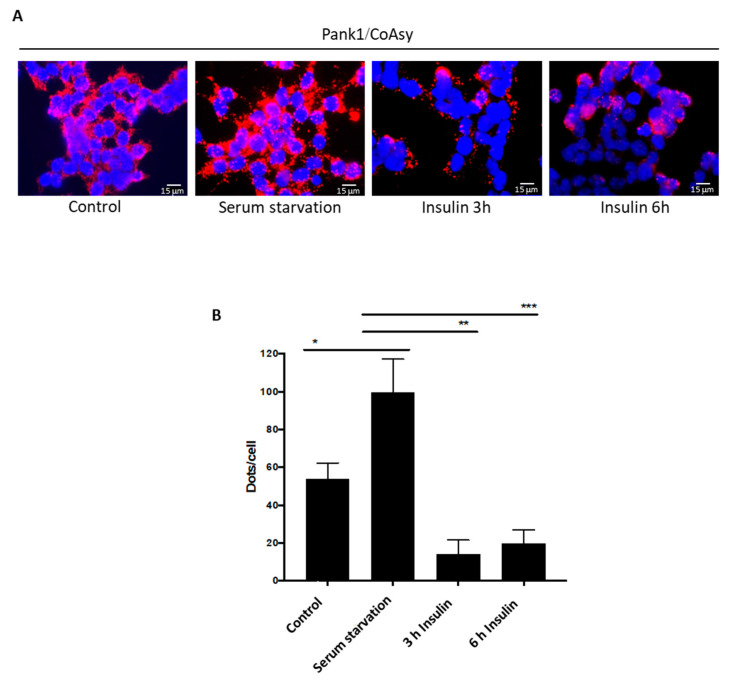
Insulin treatment inhibits the interaction between Pank1 and CoAsy in serum-starved HEK293/Pank1β cells. (**A**) Pank1 and CoAsy interaction is inhibited in response to insulin treatment in HEK293/Pank1β cells. Cells were starved for 16 h and treated with 1 μΜ insulin for 3 h or 6 h. Control cells were not starved or treated with insulin. Nuclei are stained with DAPI (blue); proximity ligation assay (PLA) was performed for Pank1 and CoAsy (Red). Scale bars: 15 μm. Data are representative of three independent experiments. (**B**) Relative quantitation of Pank1/CoAsy association in tested conditions from (**A**). Dots/cell counted. Data are presented as mean ± standard error of the mean (SEM) from *n* = 3 experiments (* *p* < 0.05; ** *p* < 0.01; *** *p* < 0.001).

Insulin is a hormone which signals nutrient availability and activates a signaling cascade that promotes the uptake of glucose, fatty acids, and amino acids into cells. It was previously shown that CoA levels are reduced in response to insulin, glucose, pyruvate, and fatty acids. We were therefore interested to examine the effect of insulin on modulating the interaction of CoA biosynthetic enzymes using the PLA assay. HEK293/Pank1β cells were seeded onto coverslips and allowed to grow for 24 h in full DMEM medium, reaching ~80% confluency. The cells were serum-starved for 16 h, and 1 μM insulin was then added for 3 h or 6 h. Control cells were grown in full DMEM medium and were not treated with insulin. Cells were fixed and the PLA assay was performed using anti-Pank1 and anti-CoAsy antibodies. The data in Figure 4A show a basal fluorescent signal in control cells and a significant increase after serum starvation, which is in line with our previous results (Figure 2 and Figure 3).

Interestingly, the signal is completely reversed by insulin treatment. Quantitation of the signal intensity confirms that both 3 h and 6 h insulin treatment reduces the fluorescent signal intensity, below that of the basal level measured in control cells (Figure 4B). These results indicate that insulin treatment induces the dissociation of enzymes which medaite the first and last steps of the CoA biosynthetic pathway, Pank1 and CoAsy.

### 2.5. Assembly of Endogenous CoA Biosynthetic Enzymes is Induced in A549 Cells in Response to Oxidative Stress

The concerted use of HEK293/Pank1β cells and PLA assay proved to be an efficient approach for testing our initial hypothesis, which revealed the association of CoA biosynthetic enzymes in exponentially growing cells and in response to oxidative stress. We next aimed to validate our results by using a different cell line, which expresses endogenous levels of proteins involved in CoA biosynthesis. Human non-small-cell lung cancer cell line A549 has been extensively used in various fields of cancer research.

**Figure 5:**
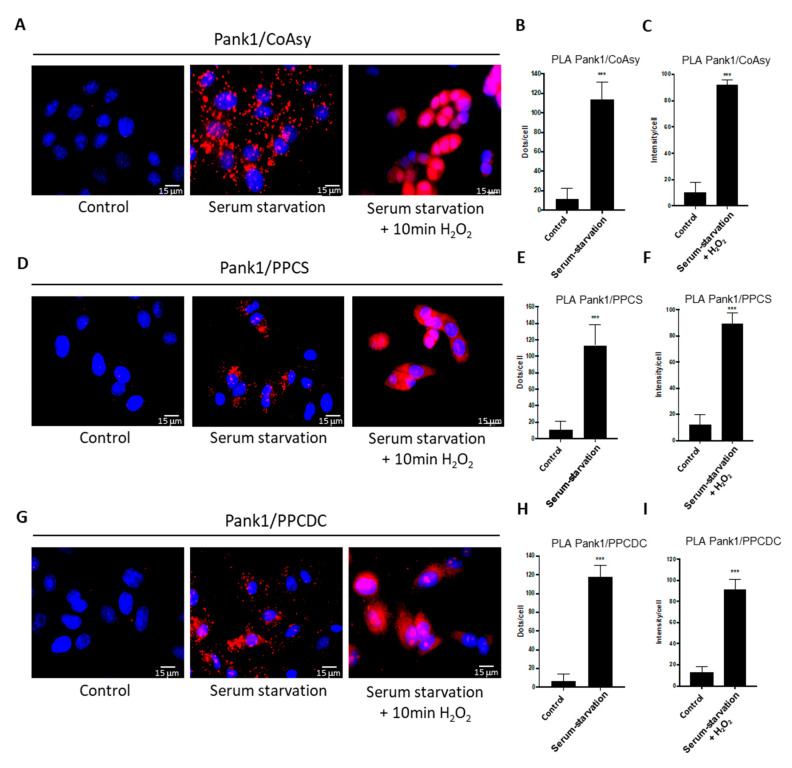
Asociation between endogenous enzymes of the CoA biosynthetic pathway is induced in A549 cells in response to serum starvation and oxidative stress. (**A**,**D**,**G**) Pank1 interacts with CoAsy, PPCS and PPCDC in A549 cells in response to serum starvation for 24 h or serum starvation followed by 10 min treatment with 500 μΜ H_2_O_2_. Nuclei are stained with DAPI (blue); proximity ligation assay (PLA) was performed for Pank1 and other enzymes of the CoA biosynthesis (Red). PLA images are representative of three independent experiments. (**B**,**E**,**H**) Relative quantitation of fluorescent dots/cell of Pank1/CoAsy, Pank1/PPCS and Pank1/PPCDC assembly in control untreated and serum starved A549 cells from (**A**,**D**,**G**). Data are presented as mean ± standard error of the mean (SEM) from *n* = 3 experiments (*** *p* <0.001). (**C**,**F**,**I**) Relative quantification of the mean fluorescence intensity per cell of Pank1/CoAsy, Pank1/PPCS and Pank1/PPCDC for control untreated and serum starved + H_2_O_2_ treated cells from (**A**,**D**,**G**). Data are presented as mean ± SEM from *n* = 3 experiments (*** *p* < 0.001).

In this study, A549 cells were seeded onto coverslips and grown in full DMEM medium for 24 h. The medium was then changed to serum-free DMEM to induce starvation stress, while control cells were grown in full medium. To further induce oxidative stress, cells incubated in serum-free medium for 24 h were treated with 500 μM H_2_O_2_ for 30 min. Cancer cells are known to be more robust and exhibit a higher resistance to oxidative stress [35]. We therefore applied a longer serum starvation and H_2_O_2_-treatment period compared to HEK293/Pank1β cells (24 h instead of 16 h, and 30 min instead of 10 min, respectively). Following treatments, cells were fixed and the PLA assay was performed using anti-Pank1 and anti-CoAsy (Figure 5A–C), anti-Pank1 and anti-PPCS (Figure 5D–F), and anti-Pank1 and anti-PPCDC antibodies (Figure 5G–I). Contrary to HEK293/Pank1β cells, no fluorescent signal appears in exponentially growing A549 cells, indicating that there is no significant association between the enzymes of the CoA biosynthetic pathway in control conditions. However, serum starvation resulted in the appearance of a large number of fluorescent dots, and a further increase in fluorescence is observed after serum starvation and H_2_O_2_ treatment, which results in signal saturation (Figure 5A,D,G). Quantitation of the assembly between Pank1 and CoAsy (Figure 5B,C), Pank1 and PPCS (Figure 5E,F), and Pank1 and PPCDC (Figure 5H,I) shows an approximate 10-fold increase in the association between these enzymes after stress treatments, compared to control conditions. The results suggest that in exponentially growing A549 cells, there is a limited interaction between enzymes of the CoA biosynthetic pathway (below the level of the PLA assay sensitivity), whereas external challenges in the form of serum starvation and oxidative stress significantly promote their interaction, leading to the association of CoA biosynthetic enzymes.

### 2.6. Growth Signaling Inhibits the Assembly of Endogenous CoA Biosynthetic Enzymes in A549 Cells

**Figure 6:**
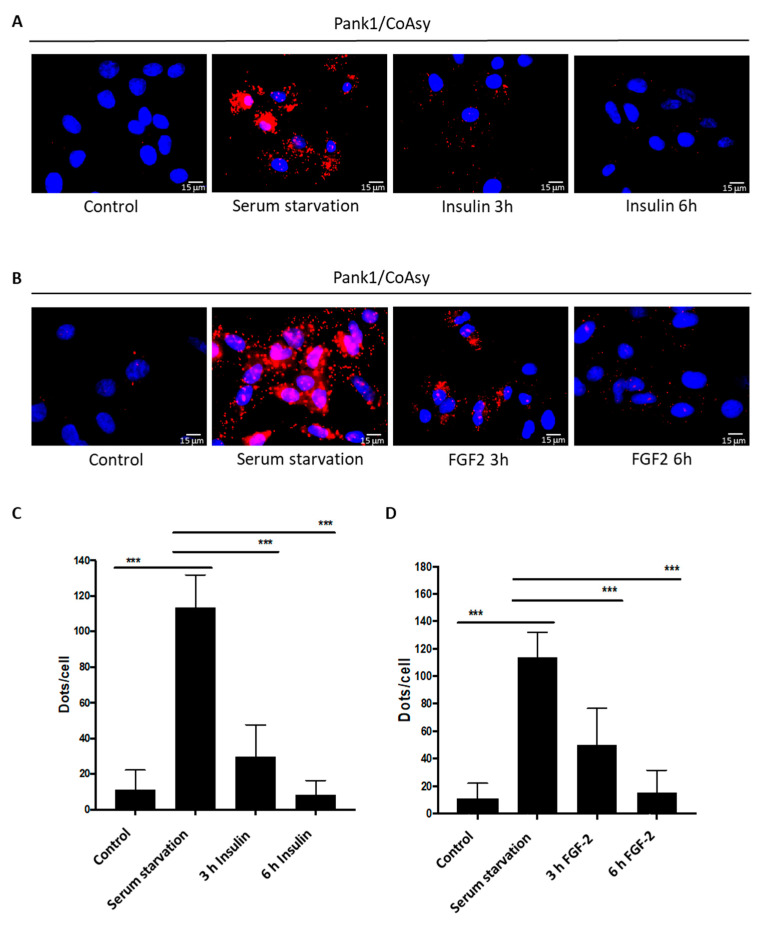
Insulin and fibroblast growth factor 2 (FGF-2) inhibit the interaction between Pank1 and CoAsy in A549 cells. Pank1 and CoAsy interaction is inhibited in response to insulin (**A**) and FGF-2 (**B**) treatment in A549 cells. Cells were starved for 24 h and treated with 1 μΜ insulin or 100 ng/mL FGF2 for 3 h or 6 h. Control cells were not starved or treated. Nuclei are stained with DAPI (blue); proximity ligation assay (PLA) was performed for Pank1 and CoAsy (Red). Scale bars: 15 μm. Data are representative of three independent experiments. (**C**,**D**) Relative quantitation of Pank1/CoAsy assembly in control, serum starved, and insulin or FGF-2 treated A549 cells from (**A**,**B**). Dots/cell counted. Data represent mean ± SEM from *n* = 3 experiments (*** *p* < 0.001).

The A549 cell line has been used to study signaling pathways and cellular functions in response to various extracellular stimuli, including insulin and growth factors. FGF-2 was shown to promote pro-survival signaling in A549 cells, while the effect of insulin on glucose and lipid metabolism in cells expressing insulin receptors (including A549 cells) is well-known [36,37,38]. Therefore, we examined the effect of insulin and FGF-2 on the association of the CoA biosynthetic enzymes in A549 cells. Cells of ~80% confluency were exposed to serum starvation for 24 h, and then treated with 1 μM insulin or 100 ng/mL FGF-2 for 3 h or 6 h. Control cells were grown in full DMEM medium and were not treated with insulin or FGF-2. The PLA assay was performed using anti-Pank1 and anti-CoAsy antibodies as described in Section 4, and cells were imaged with a fluorescent microscope. No interaction is detected between Pank1 and CoAsy in control cells, whereas serum starvation for 24 h amplifies the signal intensity ~10-fold (Figure 6), which is consistent with our previous results (Figure 5). Notably, treatment of serum-starved A549 cells with insulin (Figure 6A), or FGF-2 (Figure 6B), significantly decreases the fluorescent signal. Quantitation of the signal reveals that this reduction is time-dependent, as the number of dots per cell after insulin or FGF-2 treatment decreases after 3 h compared to serum-starved cells, and reaches that of the control cells after 6 h (Figure 6C,D). These data indicate that treatment of serum-starved A549 cells with insulin and FGF-2 leads to the dissociation of the enzymes of CoA biosynthesis.

## 3. Discussion

CoA is an essential cofactor present in all domains of life. The discovery of CoA by F. Lipmann in the middle of the last century uncovered its critical role in cellular metabolism and warranted a Nobel Prize. In the following years, numerous CoA derivatives were identified. It is now recognized that metabolically active CoA derivatives (acetyl-CoA, malonyl-CoA, HMG-CoA and succinyl-CoA, among others) are at the center of cellular metabolic and signaling pathways, mediating the citric acid cycle, the synthesis of amino acids and fatty acids, the degradation of fatty acids, protein and histone acetylation, gene regulation, neurotransmitter synthesis, and PTMs [1]. Furthermore, CoA has been recently found to function as an important antioxidant in cellular response to oxidative and metabolic stress, mediated by protein CoAlation [5,6]. As crucial parts of cellular metabolic and gene expression pathways, CoA and its derivatives are strictly maintained at levels controlled by nutrients, hormones, metabolites, and various cellular stresses [19,20,21]. Dysregulation of their biosynthesis and homeostasis has deleterious consequences and was noted in a range of pathological conditions, including cancer, metabolic disorders, and cardiac hypertrophy [23,24,27]. In addition, mutations in genes encoding for Pank2 and CoAsy were found to cause the severe neurodegenerative disorder NBIA [25,26].

**Figure 7:**
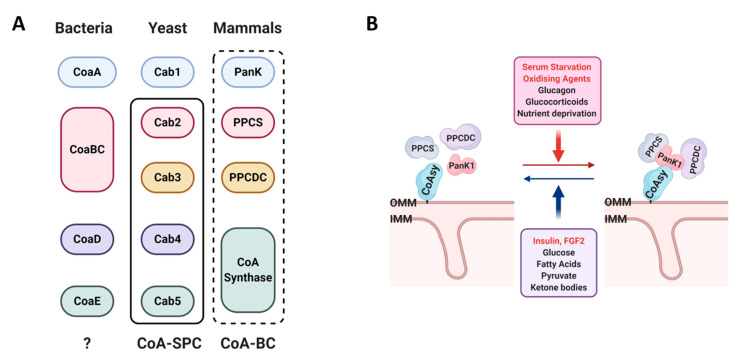
(**A**) Key players of the CoA biosynthetic pathway in bacteria, yeast and mammals. Solid rectangle indicates components of the CoA-synthesizing protein complex (CoA-SPC) described in yeast [18]. Dotted rectangle indicates a potential CoA biosynthetic complex (CoA-BC) in mammalian cells. (**B**) Negative and positive regulators of CoA biosynthesis in mammalian cells. Apart from the outer mitochondrial membrane (OMM), CoAsy was found to be localized in the mitochondrial matrix and nucleus [18,26,30,31].

A CoA biosynthetic complex composed of Cab2, Cab3, Cab4, and Cab5 (analogous to PPCS, PPCDC, and CoAsy, respectively, Figure 7A) was previously described in yeast [28]. This study used co-IP to identify the associated enzymes, which suggests that they form relatively strong interactions in the complex, with the exception of Cab1 (analogous to Pank), which was not detected. Cab1 might form weaker interactions when compared to the other enzymes, and dissociate from the complex during cell lysis and IP. Here, we investigated the existence of a CoA biosynthetic complex in mammalian cells using commonly employed conventional techniques such as co-IP and gel filtration followed by WB analysis, but our efforts were unsuccessful. The in situ PLA method allowed us to demonstrate specific interactions between enzymes of the CoA biosynthetic pathway in exponentially growing cells, as schematically presented in Figure 7A. We further reveal that the association of the CoA biosynthetic enzymes is strongly upregulated by serum starvation and oxidative stress, whereas insulin and FGF-2 promote their dissociation (Figure 7B). Many essential metabolic enzyme complexes employ the process of substrate channeling to achieve efficient catalysis, by siphoning the substrates and intermediates between their active sites. Among them are pyruvate dehydrogenase, which produces acetyl-CoA from pyruvate, and fumarase, which catalyzes the seventh reaction of the citric acid cycle [39]. The clustering of enzymes to form a CoA-synthesizing complex provides many structural and functional benefits over individual enzymes acting separately. It may allow for the five consecutive reactions of CoA synthesis to proceed efficiently and without the intermediates diffusing from the biosynthetic complex.

The mitochondrial pool of CoA (2.2–5 mM) is significantly higher than the cytosolic CoA pool (20–140 μM) [40,41,42]. All enzymes of the CoA biosynthetic pathway are found in the cytosol, with the exception of the Pank2 isoform which is localized in mitochondria. The subcellular localization of CoAsy is controversial, as it was reported to be localized in the mitochondrial matrix [26,30], outer mitochondrial membrane [18], and nucleus [31]. CoAsy was found to be anchored to the OMM via its N-terminal hydrophobic region with both enzymatic domains facing the cytosol [18]. We therefore hypothesize that CoAsy can function as a scaffold protein in regulating the assembly of CoA biosynthetic enzymes (Figure 7B). It was previously shown that CoAsy activity is strongly upregulated by phosphatidylcholine and phosphatidylethanolamine, which are the main components of the OMM [18]. The cytosolic localization of individual enzymes allows for their regulation by external stimuli such as growth factor, nutrient, and stress signaling. In response to these signals, clustering of the CoA biosynthetic enzymes around CoAsy at the OMM may allow efficient catalysis involving substrates and intermediates channeling, as well as the availability of the produced CoA to mediate both cytosolic and mitochondrial processes. The biosynthesis of CoA at the OMM may result in high local concentration and efficient transport of this cofactor into mitochondria by the SLC25A42 transporter [43]. In addition, CoA is essential for the transport of fatty acids into mitochondria, where they are used to generate energy via β-oxidation. Fatty acids are activated by acyl-CoA synthetase (ACS) at the OMM, converting them to fatty-acyl CoA thioesters, which are then transported into mitochondria by the carnitine shuttle [39]. Localized CoA production at the OMM would facilitate fatty acid activation by providing ample CoA as substrate for ACS, thus aiding their transport into mitochondria and promoting the crucial reaction of β-oxidation.

The existence of a CoA biosynthetic complex in mammalian cells presents an important way to regulate the production of CoA, which is necessary for various metabolic and signaling processes in physiological conditions, and in the antioxidant response during cellular stress. The increased assembly of CoA biosynthetic enzymes during oxidative stress observed in this study correlates with the function of CoA as an important cellular antioxidant. Overall, this study advances the current understanding of CoA biosynthesis in mammalian cells. We reveal the association between the enzymes that drive CoA biosynthesis, and further show that it is upregulated in response to oxidative stress and serum starvation, and downregulated by growth factor signaling. Future research on the structural and functional characterization of the CoA biosynthetic complex, the mode of binding and specific interactions between the proteins, as well as its points of regulation in mammalian cells would be of particular interest. A low molecular weight compound, PZ-2891, that acts as an allosteric Pank activator and modulates CoA levels in vivo was recently developed, and has been explored as a therapeutic modality for pantothenate kinase associated neurodegeneration (PKAN), which is the most common form of NBIA [44]. Additionally, the existence of the CoA biosynthetic pathway across the phylum makes it a potential target for the development of antibiotics, which would target the microbial system.

## 4. Materials and Methods

### 4.1. Reagents and Chemicals

Unless Otherwise Stated, All Common Reagents Were Obtained from Merck (Dorset, UK).

#### Antibodies

Anti-CoA antibody production and characterization were previously described [45]. Anti-Pank1 mouse monoclonal antibody was obtained from Abnova (H00053354-B01P, London, UK) (the specificity on WB is shown in Supplementary Figure S2), anti-CoAsy rabbit polyclonal antibody from Sigma-Aldrich (HPA022875, Dorset, UK), anti-PPCS rabbit polyclonal antibody from Proteintech Europe (18001-1-AP, Manchester, UK), and anti-PPCDC rabbit polyclonal antibody from Abcam (ab169942, Cambridge, UK). Secondary anti-mouse antibody (Alexa Fluor 680 goat anti-mouse IgG, A28183) was purchased from Life Technologies (Loughborough, UK). For WB, anti-Pank1 antibody was diluted in Odyssey blocking buffer (1:500), and anti-mouse antibody was used at a 1:10,000 dilution in blocking buffer supplemented with 0.02% sodium dodecyl sulphate (SDS).

### 4.2. Cell Culture and Generation of the HEK293/Pank1β Cell Line

HEK293/Pank1β and A549 cells were maintained in DMEM (Lonza, Slough, UK) supplemented with 10% FBS (Hyclone, Cramlington, UK), 50 U/mL penicillin and 0.25 µg/mL streptomycin (1%, P/S) (both from Lonza). Cells were grown in 60 mm or 100 mm tissue culture dishes in a humidified incubator at 37 °C and 5% CO_2_ (they were mycoplasma-free) and were sub-cultured at 80% confluency.

HEK293/Pank1β stable cell line was generated according to the previously published protocol [3]. HEK293 cells were co-transfected at 70–80% confluency with 5.4 mg of pLEX-Pank1β or pLEX-MCS (empty vector), and 3.5 mg of pLP-1, 1.3 mg of pLP-2 and 1.8 mg of VSVG packaging plasmids using ExGene500 (Invitrogen, Inchinnan, UK). Recombinant lentiviruses were produced as recommended (Invitrogen) and stored at −80 °C. HEK293 cells were infected with 1 mL of the viral stock at 50–60% confluency, and were incubated at 37 °C, 5% CO_2_ in complete DMEM for 48 h. The culture medium was then changed to selection medium (DMEM containing 2 mg/mL puromycin) and stable cell lines were generated using puromycin selection. Stable overexpression of Pank1β was confirmed by WB using an anti-EE antibody.

### 4.3. Treatment of HEK293/Pank1β and A549 Cells

HEK293/Pank1β or A549 cells were seeded onto poly-L-lysine coated coverslips and grown for 24 h in DMEM supplemented with 10% FBS and 1% P/S. The medium was replaced with pyruvate-free DMEM (10% FBS and 1% P/S) supplemented with 5 mM glucose, and cells were incubated for a further 24 h. Media were then changed to FBS-free DMEM (containing 1% P/S), and 16 h (HEK293/Pank1β) or 24 h (A549) later, cells were treated with 500 μM H_2_O_2_ for 10 min (HEK293/Pank1β) or 30 min (A549). For insulin and FGF-2 stimulation, serum-starved cells were treated with 1 μM insulin or 100 ng/mL FGF-2 for 3 h or 6 h. Control cells were grown in full media and were not treated.

### 4.4. SDS-PAGE and Western Blot Analysis

Cell lysates containing ~30 μg of protein were heated at 99 °C for 5 min in SDS- polyacrylamide gel electrophoresis (PAGE) loading buffer containing 63 mM Tris-HCl (pH 6.8), 10% glycerol, 2% SDS, 0.0025% bromophenol blue, and 100 mM dithiothreitol (DTT) at final concentration. Proteins were separated by SDS-PAGE on 4–20% precast gels, and transferred to a low-fluorescence polyvinylidene fluoride (PVDF) membrane (Bio-Rad Laboratories, Wales, UK) by WB. Bands were visualized using fluorescently labelled secondary antibodies and the Odyssey infrared imaging system (Odyssey Scanner CLx and Image Studio Lite software, LI-COR Biosciences, Cambridge, UK).

### 4.5. Proximity Ligation Assay

Proximity ligation assay (PLA) was performed using the Duolink In Situ Red Starter Kit Mouse/Rabbit (DUO92101, Sigma-Aldrich, Dorset, UK,), and all reagents used for the procedure are from this kit. Approximately 0.1 million HEK293/PanK1β or A549 cells were seeded onto poly-L-lysine coated coverslips and grown for 24 h in full medium (DMEM, FBS, P/S). Media were then changed to FBS-free DMEM, and 16 h or 24 h later cells were treated with 500 μM H_2_O_2_ for 10 min or 30 min. To promote growth factor signaling, serum-starved cells were treated with 1 μM insulin or 100 ng/mL FGF-2 for 3 h or 6 h. Control cells were grown in full media without treatment. Treated or not treated cells were fixed with 4% paraformaldehyde in Tris-buffered saline (TBS) supplemented with 0.2% tween-20, for 20 min at room temperature (RT). All incubations from this point on were carried out at 37 °C, in a dark and humid chamber. Coverslips were blocked with 1% bovine serum albumin (BSA) and then with Duolink blocking solution, both for 30 min. Anti-Pank1, anti-CoAsy, anti-PPCS, or anti-PPCDC antibodies were then added (20 μg/mL in Duolink antibody diluent), and slides were incubated for 1 h. Following incubation, the slides were washed in wash buffer (10 mM Tris, 150 mM sodium chloride (NaCl), 0.05% tween-20, pH 7.4) and then incubated with PLA PLUS and PLA MINUS probes (diluted 1:5) for 1 h. The PLA probes are secondary anti-mouse or anti-rabbit antibodies conjugated to unique DNA oligonucleotides (the sequence of which is not shared by the manufacturer). After another washing step in wash buffer, ligase was added (1:40 dilution in ligase solution), and the slides were incubated for 30 min to allow for the ligation and circularization of the DNA-oligos. Finally, the coverslips were washed with wash buffer, and incubated with DNA polymerase (1:80 dilution in amplification solution) for 100 min. Following incubation, the slides were washed again with a wash buffer without tween-20, and dried overnight. The slides were then mounted with one drop of Duolink in situ mounting medium containing DAPI to stain the nuclei, and analyzed with a fluorescent microscope (Axioskop, Zeiss).

### 4.6. Statistical Analysis

PLA images were quantified as previously described [46]. The number of red PLA dots per cell was analyzed using the BlobFinder software (Centre for Image Analysis, Uppsala University, Uppsala, Sweden): 50 square pixels was the maximum nucleus size, 100 pixels the radius for the cytoplasm and the blob-threshold was fixed at 3 to minimize background signals. The fluorescence intensity was analyzed for each image with the ImageJ 1.47v software (National Institutes of Health (NIH), Bethesda, MD, USA) and was divided by the number of DAPI nuclei per image to obtain a normalized intensity per cell. For comparison of two groups of PLA data, non-parametrical two-tailed Student’s *t*-test was used assuming unequal variances with Microsoft Excel for Mac 2011 (Version 14.0.0). Statistical significance was established for *p* < 0.05 and the statistical variability was estimated with the standard error of the mean (SEM).

## Data Availability

The data that support the findings of this study are available from the corresponding author upon reasonable request.

## Supplementary Materials

The following are available online at https://www.mdpi.com/1422-0067/22/3/1131/s1.

## Abbreviations

CoA	Coenzyme A
PanK	Pantothenate kinase
PPCS	4′-phosphopantothenoylcysteine synthetase
PPCDC	4′-phosphopantothenoylcysteine decarboxylase
CoAsy	CoA synthase
PLA	Proximity ligation assay
FGF	Fibroblast growth factor
NBIA	Neurodegeneration with brain iron accumulation
