# Supplementary material for: Regulation of the CoA Biosynthetic Complex Assembly in Mammalian Cells
Source: Int J Mol Sci. 2021 Jan 24;22(3):1131. doi: 10.3390/ijms22031131 (PMC7865483; doi:10.3390/ijms22031131)
Supplement: Supplementary file 1 [file ijms-22-01131-s001.pdf]

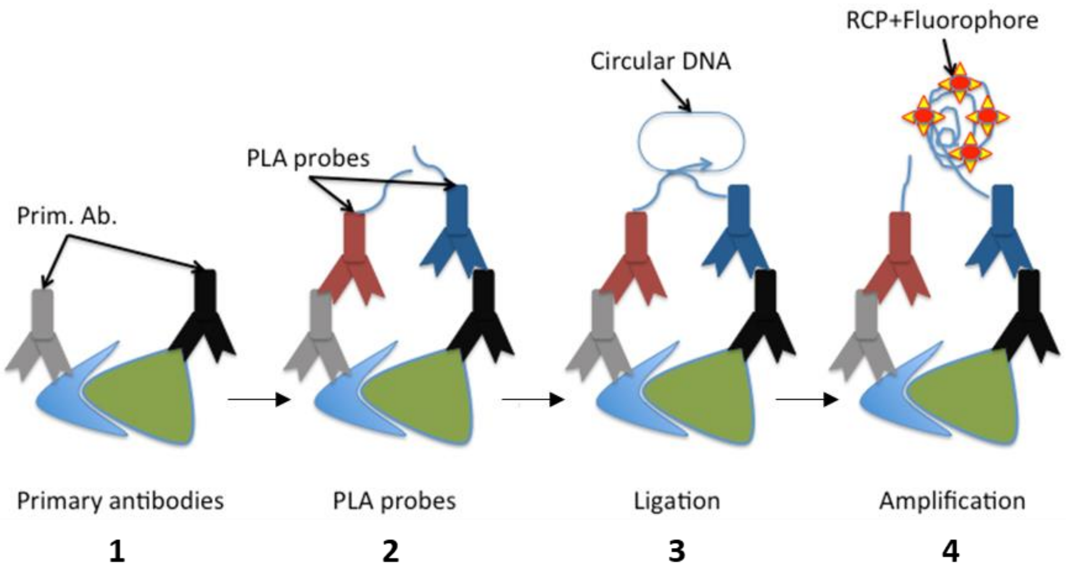

Supplementary Figure 1. Schematic diagram of the Proximity Ligation Assay (PLA). (1) In the first step, two primary antibodies raised in different species bind to the epitope on each protein of interest. (2) Secondary antibodies attached to oligonucleotides (PLA probes) are then added, and bind to the primary antibodies. (3) Two other oligonucleotides are then added together with a ligase to generate a circular DNA strand. (4) Finally, a polymerase and a fluorescent probe are added so that the polymerase amplifies the circular DNA using one of the oligonucleotides attached to the PLA probes as a primer through the rolling circle method. The fluorescent probe binds to the rolling circle product (RCP) and a red fluorescent signal is observed.

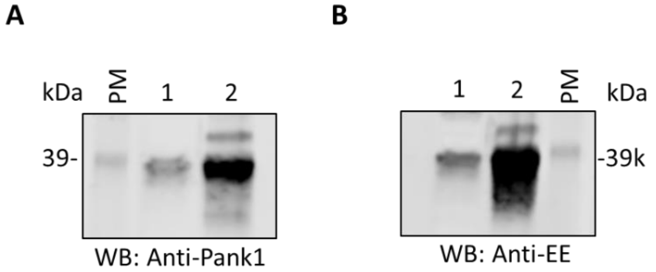

Supplementary Figure 2. Expression of EE-tagged Pank1 in exponentially growing HEK293/Pank1β cells. HEK293/Pank1β cells were lysed and EE-tagged Pank1 was immunoprecipitated (IP-ed) with anti-EE antibody. Total cell lysates (Lanes 1) and the IP-ed protein (Lanes 2) were analysed by Western Blot with anti-Pank1 (**A**) and anti-EE (**B**) antibodies. PM: protein marker.
